# Geraniol Treatment for Irritable Bowel Syndrome: A Double-Blind Randomized Clinical Trial

**DOI:** 10.3390/nu14194208

**Published:** 2022-10-10

**Authors:** Chiara Ricci, Fernando Rizzello, Maria Chiara Valerii, Enzo Spisni, Paolo Gionchetti, Silvia Turroni, Marco Candela, Federica D’Amico, Renato Spigarelli, Irene Bellocchio, Giovanni Marasco, Giovanni Barbara

**Affiliations:** 1 Department of Clinical and Experimental Sciences, University of Brescia, Spedali Civili 1, 25121 Brescia, Italy; 2 IBD Unit, IRCCS, Azienda Ospedaliero-Universitaria di Bologna, University of Bologna, Via Massarenti, 9, 40138 Bologna, Italy; 3 Department of Biological, Geological and Environmental Sciences, University of Bologna, Via Selmi, 3, 40126 Bologna, Italy; 4 Unit of Microbiome Science and Biotechnology, Department of Pharmacy and Biotechnology, University of Bologna, Via Belmeloro, 6, 40126 Bologna, Italy; 5 Division of Internal Medicine, IRCCS, Azienda Ospedaliero-Universitaria di Bologna, University of Bologna, 40126 Bologna, Italy

**Keywords:** geraniol, Irritable Bowel Syndrome (IBS), microbiota, inflammation, dysbiosis

## Abstract

Geraniol is an acyclic monoterpene alcohol with well-known anti-inflammatory and antimicrobial properties which has shown eubiotic activity towards gut microbiota (GM) in patients with irritable bowel syndrome (IBS). Methods: Fifty-six IBS patients diagnosed according to Rome III criteria were enrolled in an interventional, prospective, multicentric, randomized, double-blinded, placebo-controlled trial. In the treatment arm, patients received a low-absorbable geraniol food supplement (LAGS) once daily for four weeks. Results: Patients treated with LAGS showed a significant reduction in their IBS symptoms severity score (IBS-SSS) compared to the placebo (195 vs. 265, *p* = 0.001). The rate of responders according to IBS-SSS (reduction ≥ 50 points) was significantly higher in the geraniol vs placebo group (52.0% vs. 16.7%, *p* = 0.009) mainly due to the IBS mixed subtype. There were notable differences in the microbiota composition after geraniol administration, particularly a significant decrease in a genus of *Ruminococcaceae, Oscillospira* (*p* = 0.01), a decreasing trend for the *Erysipelotrichaceae* and *Clostridiaceae* families (*p* = 0.1), and an increasing trend for other *Ruminococcaceae* taxa, specifically *Faecalibacterium* (*p* = 0.09). The main circulating proinflammatory cytokines showed no differences between placebo and geraniol arms. Conclusion: LAGS was effective in treating overall IBS symptoms, together with an improvement in the gut microbiota profile, especially for the IBS mixed subtype.

## 1. Introduction

Functional gastrointestinal disorders, now termed disorders of gut-brain interaction (DGBI) are highly prevalent disorders worldwide. Among DGBI, irritable bowel syndrome (IBS) is one of the most common, with a reported pooled prevalence of 4.1% worldwide using Rome IV criteria [1].

IBS is a chronic and debilitating disorder, characterized by recurrent abdominal pain associated with defecation or a change in bowel habits [2]. There is a limited response to currently available treatment options for IBS, including lifestyle and dietary adjustments, psychological therapy, fiber supplementation, and pharmacological therapy. In most cases, an effective treatment approach requires a combination of pharmacological and non-pharmacological approaches targeting the multiple symptoms of IBS [3,4]. IBS pathophysiology includes changes in intestinal motor function, visceral hypersensitivity, increased intestinal permeability, low-grade inflammation, and changes in the gut microbiota (GM) [5]. Several studies indicate that low-grade inflammation occurs in IBS patients due to imbalanced cytokine signaling that could involve several interleukins (IL) and chemokines, and mediators released by activated mast cells, including, histamine, proteases, and prostaglandins [6]. On the other hand, many authors have stressed the important relationship between GM changes and IBS [7]. Key findings include a decreased relative abundance of *Lactobacillus, Bifidobacterium,* and other butyrate producers, as well as increased proportions of potential pathobionts, such as *Streptococcus and Ruminococcus* [8,9,10]. GM alterations may contribute to the pathogenesis of IBS through modifications of gut physiology, including permeability, the mucosal immune system as well as modifications of mood and behavior, through the so-called microbiota-brain axis. Thus, it is reasonable to speculate that a sort of self-sustaining inflammatory loop between GM and low-grade gastrointestinal inflammation exists in IBS patients [5,10]. In this scenario, therapeutic agents capable of modulating GM have been extensively investigated in IBS [11,12,13,14]. For instance, probiotics could modulate GM, and improve bowel movement frequency, bloating, pain, and flatulence, but it is not yet clear in which cases they may be useful and in what form, dose, combination, or strain [15,16]. As for prebiotics, the few clinical trials conducted have shown an improvement in overall symptoms but a worsening in bloating, probably because of an increase in fermentative processes occurring in the colon [17,18]. Fecal microbiota transplantation (FMT) is also considered a therapeutic option for refractory IBS [19], especially for post-infective IBS where a pronounced dysbiosis is present [20].

Essential oils (EOs) have been recognized as potential treatment options for IBS, due to their ability to modulate GM [21,22]. Geraniol is a naturally acyclic monoterpene component of EOs extracted from lemongrass, rose, and other aromatic plants. Several studies on the biological activities of geraniol have shown that it is a highly active antimicrobial compound with antioxidant and anti-inflammatory properties [23,24]. Geraniol antimicrobial activities do not appear to have specific targets; like other EO components, geraniol is a hydrophobic compound capable of binding to the bacterial cell wall and modifying its dynamic organization, with consequent loss of ions and ATP depletion [25,26]. Human pathogenic bacteria are more sensitive to geraniol than commensal species although the nature of this selectivity remains unclear [24].

In a recent in vivo study on a dextran sulfate sodium (DSS)-induced colitis mouse model, orally administered geraniol (30 and 120 mg/kg die) strongly improved colitis and significantly reduced dysbiosis and cyclooxygenase-2 (COX-2) expression in the gut wall [27]. These results are in line with those obtained by Medicherla et al. [28], who found significantly reduced inflammation in colon specimens of colitic mice after oral administration of geraniol (50 and 100 mg/kg die). We previously conducted a pilot study on IBS patients to assess the anti-inflammatory and anti-dysbiotic properties of low-absorbable geraniol (8 mg/kg die) [29]. According to our findings, orally administered microencapsulated geraniol was a potent modulator of GM and reduced the Visual Analogue Scale for IBS (VAS-IBS) score, improving the quality of life of these patients. The aim of the present study was to conduct a placebo-controlled study to assess the efficacy of a low-adsorbable geraniol food supplement (LAGS) in the treatment of patients with IBS using a validated composite score for assessing IBS symptoms, together with the assessment of GM and inflammatory cytokines.

## 2. Materials and Methods

### 2.1. Study Design and Population

The study was an interventional, prospective, multicentric, randomized, double-blinded, placebo-controlled trial. All subjects who met the eligibility criteria received a 4-week treatment with LAGS or placebo. Eligibility criteria for study inclusion included subjects aged 18 to 65 years, IBS diagnosis based on Rome III Criteria, and BMI (kg/m^2^)  <  27 with a weight between 48 and 104 kg. Exclusion criteria were, intolerance to lactose or known food allergies, concomitant treatment with non-steroidal anti-inflammatory drug antibiotics, and consumption of functional food, food supplements, probiotics, and prebiotics within two months prior to the screening visit. The assumption of other IBS therapies was considered an exclusion criterion from the study. Women in pregnancy and lactation, subjects with a diagnosis of inflammatory bowel disease or celiac disease were also excluded, together with subjects with food allergy to geraniol and/or soya, subjects with serious concomitant diseases that, according to the investigator, precluded the patient’s participation in the study and subjects in other experimental drug treatments within two months prior to the screening visit. Any other inflammatory condition was excluded in these patients by C-reactive protein (CRP) and cell blood count (CBC), routinely performed as per clinical practice. Patients were asked to maintain their normal diet during the trial. Consumption of functional food and/or food supplements (including probiotics and prebiotics) was considered a drop-out criterion. Patients were informed of the full nature and purpose of the study and provided written informed consent before entering the trial. The study was conducted in conformity with the principles of the Declaration of Helsinki and Good Clinical Practice. The sites involved in enrolment and data collection were the Department of Medical and Surgical Sciences at S. Orsola University Hospital, Bologna, Italy, and the Gastroenterology Unit at Spedali Civili di Brescia Hospital, Brescia, Italy. The study was approved by each local hospital Ethics Committee (approved by the Regional Ethics Committee AVEC of the Sant’Orsola Hospital CE code 397/2018/Sper/AOUBo approved on 18 July 2018 and the Ethics Committee of the ASST Spedali Civili di Brescia: CE code NP3011 approved on 3 April 2018). The trial was retrospectively registered (registration n°: ISRCTN47041881).

### 2.2. Treatment and Randomization

The food supplement was provided in 470 mg capsules composed of 450 mg of a patented low-absorbable geraniol formulation called BIOintestil^®^ (90 mg of Palmrose EO high geraniol absorbed on 360 mg of pulverized *Zyngiber officinalis* root, European patent EP3097921), and excipients (10 mg vegetal magnesium stearate and 10 mg silicon dioxide). BIOintestil^®^ formula has been studied and patented to absorb geraniol into the ginger fibers to minimize its intestinal absorption, and deliver 85% of this monoterpene to the colon, where most of the GM resides [30]. The placebo was provided in 470 mg capsules composed of corn starch (450 mg) and excipients (10 mg vegetal magnesium stearate and 10 mg silicon dioxide). BIOintestil^®^ and placebo capsules were taken once daily, with meals for 4 weeks. The dosage of the supplement was calculated based on body weight as described in Table 1.

This dosage is lower than those previously used in the pilot study since LAGS minimizes the intestinal absorption of geraniol [30]. To minimize bias, the patients, investigators, staff, and sponsors were blinded until the end of the study. Active food supplements and placebo capsules and packages were confirmed to be indistinguishable by the external producer (Laboratorio Terapeutico MR, Florence, Italy) before the study. Starting from a randomization list centrally generated with a 1:1 scheme, each experimental site received a numbered sequence of sealed envelopes containing the assignment code.

### 2.3. Study Outcomes

The aim of the study was to evaluate symptom improvements and microbiota modulation in patients with IBS treated with LAGS compared to placebo. The primary outcome of the study was the assessment of geraniol efficacy over placebo for global IBS symptom relief according to the IBS Symptom Severity Score (IBS-SSS) dichotomous definition of responders (see below). The secondary outcomes of the study consist of the assessment of GM and inflammatory and permeability markers variations after LAGS treatment compared to the placebo.

### 2.4. Study Visits

The study consisted of two visits. During Visit 1 (V1), after eligibility evaluation and informed consent signature, patients underwent a medical examination with vital signs and symptoms evaluation. For each patient, a complete medical history, including current drug intake was obtained. Patients were then asked to complete the IBS-SSS questionnaire for assessing the presence and severity of symptoms. Briefly, IBS-SSS is a composite validated questionnaire evaluating and scoring five domains, namely abdominal pain, number of days with abdominal pain, bloating/distension, satisfaction with bowel habits, and impact of IBS on daily activities. Each measure is rated from 0 to 100, with total scores ranging from 0 to 500. A reduction in IBS-SSS score ≥ 50 points was considered to represent a clinically significative improvement (responders) as previously indicated [31]. During the V1 visit, a stool sample and two blood samples were collected for GM, cytokine, blood chemistry, and CBC analyses. During the second visit (V2) after 4 weeks of treatment (±7 days), patients were interviewed, and adverse events and concomitant therapies were recorded. Patients underwent a medical examination with vital signs and symptom evaluation including the IBS-SSS questionnaire. A stool sample and two blood samples were collected at V2 for microbiota, cytokine, blood chemistry, and blood count (CBC) analyses. Patients returned the food supplement package and compliance to therapy was evaluated by pill counting. Adverse events were also assessed at V2. All data were recorded in a case report form.

### 2.5. Gut Microbiota and Inflammatory Evaluations

Blood and stool samples were collected and analyzed in the laboratories of the University of Bologna. For GM profiling, microbial DNA was extracted from 250 mg of each fecal sample using the repeated bead-beating protocol, as previously reported [32]. The V3–V4 hypervariable regions of the 16S rRNA gene were amplified using the universal primers 341F and 785R with Illumina adapter overhang sequences, and libraries were purified and indexed according to manufacturer’s instructions (Illumina, San Diego, CA, USA). The denatured and diluted 5 pM pool was sequenced on an Illumina MiSeq platform using the 2 × 250 bp paired-end protocol as per the manufacturer’s protocol (Illumina). Sequence reads were deposited in the National Center for Biotechnology Information Sequence Read Archive (NCBI SRA; BioProject ID PRJNA852514). All sequence data were processed using a pipeline combining PANDASeq [32] and QIIME 2 [33]. Briefly, quality-filtered reads were binned into Amplicon Sequence Variants (ASVs) using DADA2 [34]. Singletons and chimeras were removed during sequence processing. Taxonomy assignment was performed using the VSEARCH algorithm [35] against the Greengenes database. Alpha diversity was computed using Faith’s phylogenetic diversity (Faith’s PD) index and the number of observed ASVs. Beta diversity was estimated by calculating the Bray-Curtis distances between the genus-level microbial profiles, which were then used as input for Principal Coordinates Analysis (PCoA) plots.

Blood for inflammatory and permeability marker analysis was collected in sodium citrate and centrifuged at 3000 rpm for 7 min. Plasma was collected and stored at −80 °C until analysis. In plasma, circulating cytokines and chemokines were quantified by using a customized detection panel purchased from Bio-techne (Minneapolis, MN, USA). The cytokines and chemokines evaluated were IL-1β, IL-4, IL-5, IL-6, IL-8, IL-10, IL-12, IL-17A, IFN-γ, TNF-α, MCP-1, MIP-1β and CCL28. The assays were performed in 96-well filter plates by multiplexed Luminex^®^-based immunoassay following the manufacturer’s instructions, as previously described [36]. Microsphere magnetic beads coated with monoclonal antibodies against the different target analytes were added to the wells. After a 30 min incubation, the wells were washed, and biotinylated secondary antibodies were added. After incubation for 30 min, beads were washed and then incubated for 10 min with streptavidin-PE conjugated to the fluorescent protein, phycoerythrin (streptavidin/phycoerythrin). After washing, the beads (a minimum of 100 per analyte) were analyzed in the BioPlex 200 instrument (BioRad^®^, Hercules, CA, USA). Sample concentrations were estimated from the standard curve using a fifth-order polynomial equation and expressed as pg/mL after adjusting for the dilution factor (Bio-Plex Manager software 5.0). Samples below the detection limit of the assay were recorded as zero, while samples above the upper limit of quantification of the standard curves were assigned the highest value of the curve. The intra-assay CV averaged 15%. Zonulin detection was performed using ELISA Kit (Cusabio, Houston, TX, USA) following the manufacturer’s instructions (detection range: 0.625–40 ng/mL; sensitivity: 0.156 ng/mL), as previously described [37]. Each sample was analyzed in duplicate and reported as picograms of zonulin per ml of plasma.

### 2.6. Statistical Analyses

Considering an expected reduction of symptoms in about 30% of subjects, as previously reported by the reduction in VAS score of the LAGS group compared to placebo in the study by Rizzello et al. [29], with an alpha-error = 0.05 and a statistical power = 0.8, a sample size of 90 patients (45 in each study arm) was calculated to evaluate geraniol clinical efficacy. For clinical and biochemical parameters, continuous variables were reported as mean and standard deviation (SD) or median and inter-quartile range (IQR), while categorical variables as number and percentage. The normality of distribution was verified with the D’Agostino–Pearson and Shapiro–Wilk tests and the homogeneity of variances (homoscedasticity) with the F-test. Variables were compared between placebo and treatment arms at V1 = baseline and V2 = follow-up (after 4 weeks from the start of treatment) and in each treatment arm at V1 vs. V2, using the Student T-test, U-Mann–Whitney test, Chi2, or Fisher’s exact tests, when appropriate. The probability values were two-sided; a probability value (*p*) less than 0.05 was considered statistically significant. Statistical analysis was performed with STATA 13.0 (College Station, TX: StataCorp LP).

As for GM, statistical analyses were carried out with the R software. The PCoA graphs and the Adonis test (permutation test with pseudo-F ratio) were made using the “vegan” package [38]. For taxonomic and alpha diversity comparisons, the Kruskal–Wallis test followed by post-hoc Wilcoxon tests (paired or unpaired as needed) was used. *p* values were corrected for multiple comparisons using the Benjamini–Hochberg method. A false discovery rate (FDR) ≤ 0.05 was considered statistically significant. FDR ≤ 0.1 was considered a trend.

## 3. Results

### 3.1. Clinical Characteristics and Outcomes

Between February 2018 and June 2021, 56 eligible patients were randomly assigned to the placebo group (*n* = 27) or the LAGS group (*n* = 29). Five patients, 3 in the placebo arm (11%) and 2 in the treatment arm (7%)] were lost to follow-up, therefore they did not carry out V2, due to personal or SARS-CoV-2 pandemic-related problems. We registered only two adverse events (AEs) in the treatment arm, of which only one was potentially related to the intake of geraniol since reporting unspecified gastric symptoms (1/25, 4%), and none in the placebo arm. A larger statistical sample would also allow a better assessment of any adverse effects. The baseline patient characteristics are shown in Table 2.

**Figure 1:**
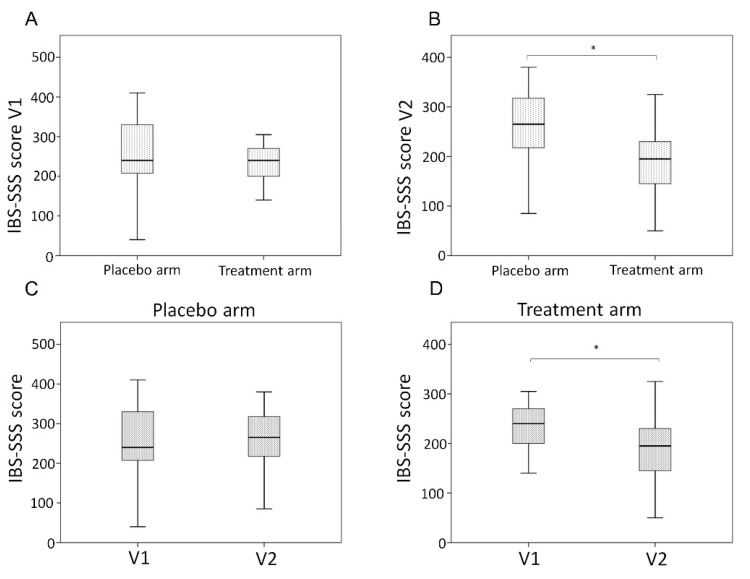
IBS-SSS at baseline (**A**), follow-up (**B**), and longitudinal evaluation according to study groups: placebo (**C**) and treatment arm (**D**). * *p* < 0.05.

Female patients accounted for 60.7% (34/56) of the total study population. The average BMI was 22.4 kg/m^2^ in the placebo arm and 22.6 kg/m^2^ in the treatment arm. At baseline, there were no differences in total and single domain IBS-SSS (Table 3 and Figure 1A). No AEs were registered during follow-up evaluations in both groups.

At the end of the study, patients in the LAGS group reported reduced intensity of abdominal pain (*p* = 0.001), days with abdominal pain (*p* = 0.032), the intensity of bloating (*p* = 0.021), and increased satisfaction with bowel habits (*p* = 0.035) compared to the placebo group (Table 3). IBS-SSS significantly decreased at V2 in the LAGS arm compared to the placebo arm (195 vs. 265, *p* = 0.001) (Figure 1B). No differences were found between the LAGS and placebo group for the interference of symptoms with daily activities. Consequently, we found a statistically significant difference in the rate of responders according to IBS-SSS (reduction of at least 50 points) in the LAGS group compared to placebo [placebo 4, (16.7%) vs. LAGS 13 (52.0%), *p* = 0.009] (Table 3). According to the IBS subtype sub-analysis, the difference in the rate of responders was statistically significant only for the IBS-M subtype [placebo 1 (7.7%) vs. LAGS group 7 (53.9%), *p* = 0.011].

Besides, the longitudinal evaluation of IBS-SSS in each study group, confirmed the absence of statistically significant differences between V2 evaluation and baseline in the placebo arm [265 (IQR 217.5; 317.5) vs. 240 (207.5; 330), *p* = 0.703, (Figure 1C) and the significant difference between V2 evaluation and baseline in the treatment arm [195 (145; 230) vs. 240 (200; 270), *p* = 0.029] (Figure 1D).

### 3.2. Gut Microbiota Modulation

**Figure 2:**
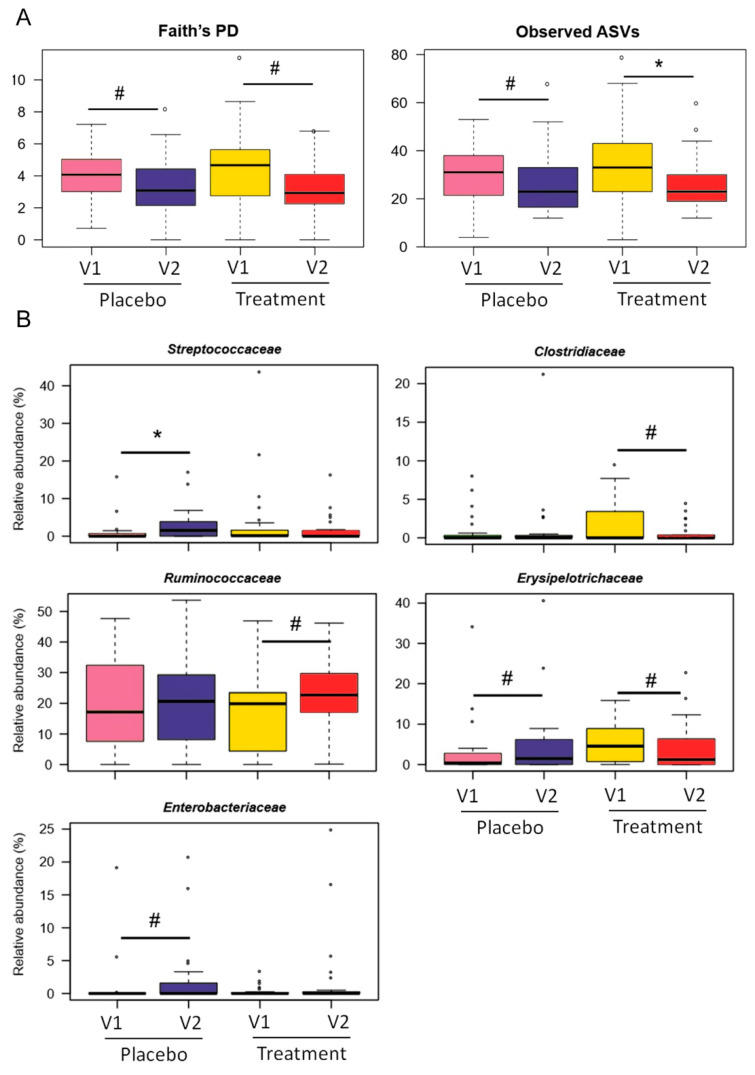

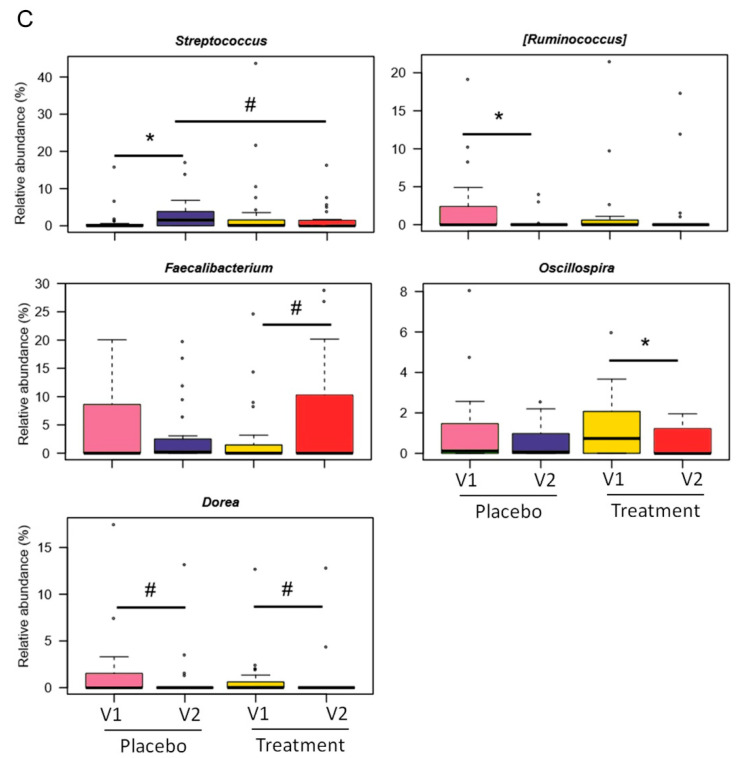
Gut microbiome profile of IBS patients after treatment with geraniol (LAGS) or placebo. (**A**) Boxplots showing the distribution of alpha diversity as measured by Faith’s PD (left) and number of observed ASVs (right) in IBS patients before (V1) and after (V2) 4 weeks of treatment with geraniol or placebo. Boxplots showing the relative abundance distribution of families (**B**) and genera (**C**) differentially represented between groups are reported. Wilcoxon signed-rank test, * for *p* < 0.05, # for *p* < 0.1.

The treatment impact on GM was assessed by 16S rRNA gene-based next-generation sequencing of 98 fecal samples collected at V1 and V2 from 25 IBS patients receiving LAGS, and 24 receiving placebo. A total of 3,190,703 high-quality reads (mean ± SD, 32,558 ± 14,574) were obtained and clustered into 2477 ASVs. No significant differences among groups were found for beta diversity according to the Bray–Curtis dissimilarity metric (*p* > 0.1, Adonis) (Figure S1). On the other hand, alpha diversity decreased but did not reach statistical significance over time in both treatment groups (*p* ≤ 0.1, Wilcoxon signed-rank test) (Figure 2A).

From the taxonomic standpoint (Figure S2), the GM profile of patients was overall dominated by the phylum Firmicutes (mean relative abundance across the dataset, 69.0%) with the remainder composed of Actinobacteria (15.3%) Bacteroidetes (8.4%) and Verrucomicrobia (5.1%). *Lachnospiraceae* (23.9%), *Ruminococcaceae* (20.3%), *Bifidobacteriaceae* (7.7%) and *Coriobacteriaceae* (7.6%) were the dominant families [39]. As for the treatment impact on GM composition, both common and unique microbial signatures were observed (Figure 2B,C). Among those shared, it should be noted that the proportions of *Dorea* tended to decrease over time in both groups (*p* ≤ 0.1). On the contrary, we observed a contrasting trend in the *Erysipelotrichaceae* family, whose amounts tended to decrease in the BIOintestil^®^ group and to increase in the placebo group (*p* = 0.1). With particular regard to the treatment arm, we found a trend towards an increase in the relative abundance of *Ruminococcaceae*, specifically of *Faecalibacterium* (*p* = 0.09), while a decrease of *Clostridiaceae* (*p* = 0.1) and another genus of *Ruminococcaceae*, *Oscillospira* (*p* = 0.01). On the other hand, patients assigned to the placebo group showed increased levels of the *Streptococcaceae* (*p* = 0.05) and *Enterobacteriaceae* families (*p* = 0.1) over time, and a reduction of [*Ruminococcus*] (from the *Lachnospiraceae* family) (*p* = 0.02).

**Figure 3:**
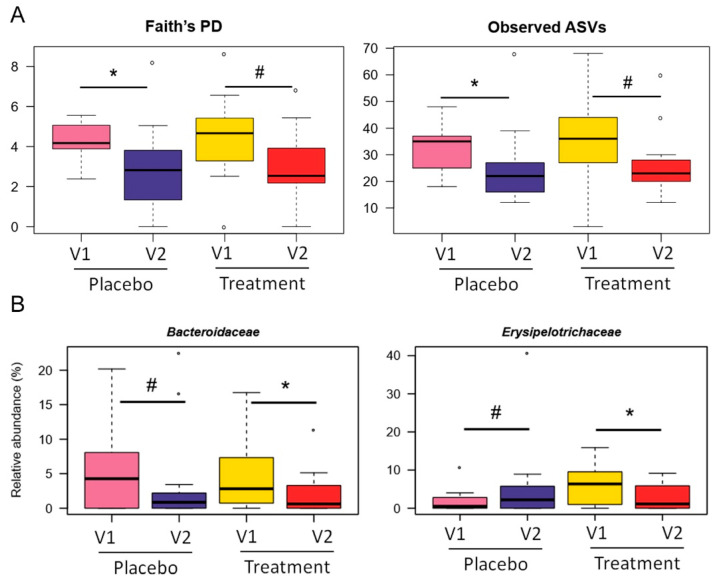

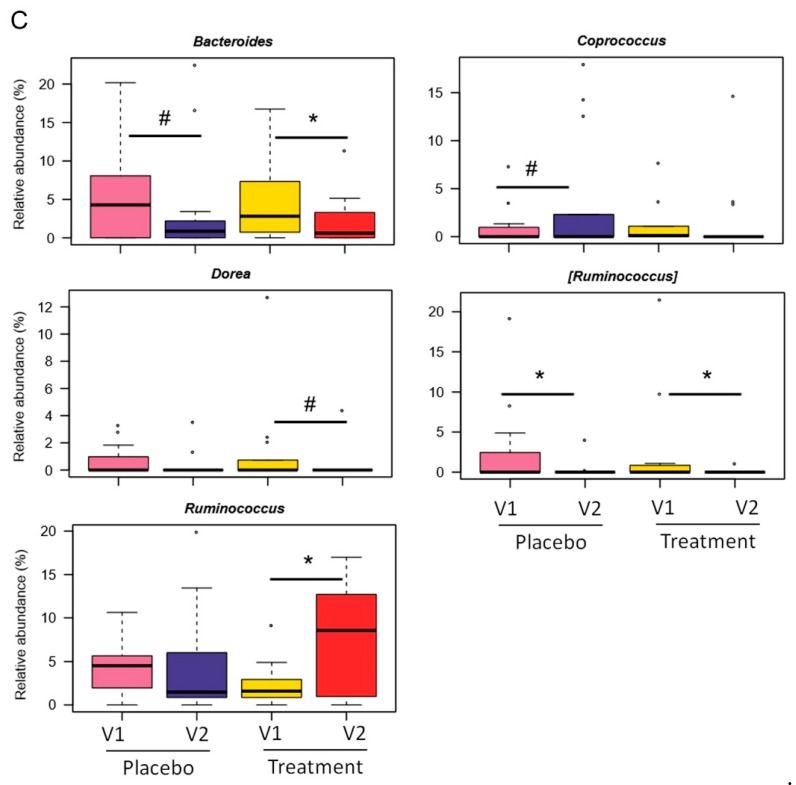
Gut microbiome profile of IBS-M patients after treatment with geraniol (LAGS) or placebo. (**A**) Boxplots showing the distribution of alpha diversity as measured by Faith’s PD (**left**) and number of observed ASVs (**right**) in IBS-M patients before (V1) and after 4 weeks (V2) of treatment with geraniol or placebo. Boxplots showing the relative abundance distribution of families (**B**) and genera (**C**) differentially represented between groups. Wilcoxon signed-rank test, * for *p* < 0.05, # for *p* < 0.1.

The analyses were then repeated only in patients with IBS-M, the subtype for which a significant reduction in IBS-SSS was shown after LAGS treatment (Figure 3). Again, no significant differences were found among groups for beta diversity (*p* > 0.1, Adonis) (Figure S1), while alpha diversity significantly decreased in the placebo group (*p* ≤ 0.05, Wilcoxon signed-rank test) but not in the LAGS group (*p* < 0.1) (Figure 3A). Taxonomically (Figure 3B,C), we confirmed the contrasting trend for *Erysipelotrichaceae*, i.e., a decrease in the treatment arm (*p* = 0.03) while an increase in the placebo arm (*p* = 0.1). Furthermore, both groups showed a reduction over time in the proportions of *Bacteroidaceae*, mainly *Bacteroides* (*p* ≤ 0.1), and *Ruminococcus* (from *Lachnospiraceae*) (*p* ≤ 0.05). As for discriminating features, the increase in *Ruminococcus* (from *Ruminococcaceae*) (*p* = 0.05) and the decrease in *Dorea* (*p* = 0.06) specifically characterized patients receiving LAGS, while those receiving placebo showed an increase in *Coprococcus* (*p* = 0.1).

### 3.3. Inflammation and Intestinal Permeability Markers

No differences were observed in the circulating levels of cytokines and permeability markers at V1 for LAGS vs. the placebo group. We analyzed the effects of geraniol on the systemic inflammatory profile of IBS patients at V2, consisting of blood IL-1β, IL-4, IL-5, IL-6, IL-8, IL-10, IL-12, IL-17A, IFN-γ, TNF-α, MCP-1, MIP-1β, and CCL28. IBS patients showed very low levels of systemic inflammation [39]. LAGS, at the dosages administered, did not significantly change their circulating levels, and the results demonstrated comparable values in the placebo and treatment arms. Circulating levels of zonulin were also not significantly affected by geraniol treatment, and its plasma concentration was very similar in the two groups, both at V1 and V2. Even restricting the statistical analysis to the IBS subgroups, no significant changes were found in these biomarkers (Figure S3).

## 4. Discussion

This is the first placebo-controlled study evaluating the effects of geraniol, delivered in a low-adsorbable form, on symptoms and GM in IBS patients. The main result of this study is the significant effect of geraniol in reducing overall IBS symptoms accompanied by an improvement in the GM profile.

For the assessment of clinical efficacy, we used IBS-SSS, which is a multidimensional tool both for selecting symptomatic patients for clinical trials and for measuring response to treatment [40]. This tool has been used in the past mainly for the assessment of cognitive and behavioral therapy for IBS, while its use for pharmacological therapies was considered within the future aims of the Rome Foundation working team [40]. We found that a statistically significant number of patients treated with LAGS reported symptom amelioration when compared to the placebo arm. As a matter of fact, the current first-line pharmacological treatment for IBS is symptoms-based and includes spasmolytic or antispasmodic agents, loperamide for diarrhea or mixed-bowel dysfunction on need-use, and dietary fiber and osmotic laxative for constipation [41,42]. Peppermint oil has been suggested as an additional first-line therapy for global symptoms and abdominal pain in IBS [43]. Nevertheless, in two randomized trials of patients with IBS, neither small-intestinal-release nor ileocolonic-release of peppermint oil produced statistically significant relief of IBS symptoms [44,45]. Moreover, known reported side effects of peppermint oil use were heartburn, dry mouth, and belching [44,45].

EOs are capable of modulating GM, as extensively reported, in animals and humans [46,47,48]. Within this pivotal trial, in addition to the GM modulation, we confirmed the results of the clinical efficacy of geraniol, previously reported by Rizzello and coauthors [29] using a mono-dimensional scale for the assessment of IBS symptoms, showing in a multidimensional fashion that LAGS administration is capable of reducing several IBS symptoms such as abdominal pain severity and frequency, and bloating, and ameliorating bowel habit satisfaction.

Regarding cytokine and permeability markers, in our previous pilot study [30] we found a significant reduction of MIP-1b, even circulating at lower doses, in peripheral blood (plasma). This observation was not confirmed in the present study. A possible explanation could be due to the fact that, while in the pilot study the formulation of geraniol was absorbed at 50% in the intestine, the LAGS used in this trial (BIOintestil^®^) has a very low intestinal absorption (around 15%), thus decreasing the amount of geraniol reaching the systemic circulation and possibly affecting cytokine levels [30].

Given the absence of differences in cytokine and permeability markers among the placebo and treatment arms, the observed clinical effects could be associated with the antispasmodic effect and some of the GM fluctuations possibly exerted by geraniol. Indeed, a previous experience using geraniol showed neuroprotective qualities in terms of reduced activation, desensitization, and deactivation of the α-amino-3-hydroxy-5-methyl-4-isoxazolepropionic acid receptor (AMPAR) [49]. AMPARs are part of the ionotropic glutamate receptor family that respond to glutamate and are responsible for most of the fastest excitatory neurotransmission [49]. However, these assumptions will have to be confirmed in future studies.

Besides, as a counterproof of GM modulation in IBS, dysbiosis can be targeted with several GM-based interventions including diet, pre- and probiotics, poorly absorbable antibiotics, or fecal microbiota transplantation (FMT) [50]. For example, a recent meta-analysis reported a significant effect on global symptoms or abdominal pain for probiotics as a group, with RR 0.78 (CI 95%: 0.63–0.95) [51]. In addition, a greater effect of rifaximin than placebo (RR 0.84; 95% CI 0.79–0.90) was reported [51]. On the other hand, no firm conclusions can be drawn according to meta-analyses evaluating the role of FMT in IBS treatment [52,53].

Within this study, we showed that geraniol may favor some GM features that are known to be associated with host homeostasis, in line with the previous pilot study [30]. In particular, we confirmed the increase in short-chain fatty acid producers belonging to the *Ruminococcaceae* family, namely *Faecalibacterium* (although the variation in the latter was only a trend). It should be remembered that *Faecalibacterium* is known to produce butyrate (a multifaceted molecule, crucial for metabolic and immunological homeostasis) [54,55], is positively associated with improved Bristol Stool Scale score [56], and is typically reduced in IBS and other intestinal and non-intestinal disorders (probably because of increased oxidative stress and decreased barrier functions) [57]. Moreover, geraniol led to a decrease in the relative abundance of *Oscillospira*, a microorganism already associated with IBS and particularly with constipation [58], as well as *Erysipelotrichaceae*, a bacterial family currently poorly characterized but generally associated with increased inflammatory tone and previously shown to be enriched in IBS [14,59]. Notably, the proportions of *Erysipelotrichaceae* tended to increase in the placebo group, parallel to the increase in other potential opportunistic pathogens, such as *Enterobacteriaceae* and *Streptococcaceae* members. Nevertheless, patients in the placebo group still experienced some beneficial changes, namely reduced proportions of the pro-inflammatory mucus degrader *Ruminococcus* (from the *Lachnospiraceae* family), whose species *R. gnavus* and *R. torques* have previously been suggested as potential biomarkers of IBS [6,60] and associated with increased symptom severity [61], probably also through impaired tryptamine production [62]. It should be noted that some of the observed differences were just trends, likely reflecting the heterogeneity of the study cohort. Interestingly, when focusing on the IBS-M subtype, for which the highest number of treatment responders was found, the contrasting pattern of *Erysipelotrichaceae* and the geraniol-related increase in *Ruminococcaceae* members (i.e., *Ruminococcus*) were confirmed. Again, some positive changes were also observed in the placebo group, such as the increase in the short-chain fatty acid producer *Coprococcus*, while the relative abundance of *Ruminococcus* decreased significantly in both groups.

The fluctuations in the GM of IBS patients, explaining our findings for the placebo group, are in line with those of a recent multi-omics longitudinal study [63] comparing the GM of IBS patients and healthy controls, which confirmed that beyond treatments, lifestyle intervention and environmental factors, the GM variability over time may also reflect changes in disease activity; in particular, some IBS subtypes as IBS-C exhibited a greater temporal variability. Unfortunately, within this trial, we could not explore psychological and dietary variations over treatment time, which may affect GM fluctuations even in the placebo group as recently suggested [6].

We are aware that our study has major limitations, such as the limited number of patients enrolled and the prevalence of the IBS-M clinical subtype, which may prevent us from extending our results to other IBS subtypes. In addition, the study was designed and registered before the release of the Rome IV criteria for IBS diagnosis; this may have additionally introduced a selection bias in the study population, even though more than three-quarters of the Rome III IBS patients can be classified as Rome IV IBS according to a recent study [64]. Nevertheless, the interim analysis showed the high efficacy of LAGS and therefore it was decided to stop the trial, also in relation to the enrollment difficulties related to the SARS-CoV-2 pandemic. On the other hand, it must be noted that we carefully selected patients, and the results in terms of improved symptomatology and reduced dysbiosis are certainly promising, especially for the IBS-M subtype, in which the LAGS effect on symptoms was statistically significant, also due to the highest number of patients included in this subgroup. Although not statistically significant, a decrease in IBS-SSS was nevertheless observed in all IBS subtypes considered. With specific regard to GM, as discussed above, the analysis on the whole non-stratified cohort is likely responsible for the non-significant trends that were observed. While this could lead to the identification of IBS subtype-independent GM features, it stresses the need for further studies in less heterogenous groups to obtain more conclusive results.

## 5. Conclusions

Geraniol in the low-adsorbable formulation called BIOintestil^®^ is effective in treating overall IBS symptoms and has the potential to improve the GM profile, particularly in the IBS-M subtype. These data are interesting because, in this subtype of IBS, some therapies cannot be used (antidiarrheals or laxatives). Since most of the patients included in this study exhibited mixed bowel habits, further studies are needed to confirm the efficacy of LAGS in other IBS subtypes. Whether our data will be further confirmed, it is possible to speculate that geraniol, in this low-adsorbable formula, can be used as a first-line treatment for IBS, especially for IBS-M patients, with both an antispasmodic and GM-modulating effect.

## Figures and Tables

**Table 1:** Dosage of LAGS capsules. Each capsule contains 450 mg of BIOintestil^®^, composed of 90 mg of Palmrose Essential Oil (75 mg geraniol) and 360 mg of pulverized *Zyngiber officinalis* root.

Patient Weight (kg)	Capsules (*n*.)
45–67	2
68–89	3
90–111	4

**Table 2:** Demographics in study cohort in IBS and placebo arms at V1 = baseline.

	Placebo N (%) or Median (IQR) *n* = 24	Treatment N (%) or Median (IQR) *n* =25	*p*
Age	40 (20–58)	30 (19–52)	0.05982
Gender (Female)	50%	72%	/
BMI	22.4 (18–26)	22.6 (18–27)	0.9597

**Table 3:** IBS-SSS items, clinical outcomes in study cohort and in IBS subtype in placebo and treatment arms (V1 = baseline, V2 = follow-up after 4 weeks from the start of treatment).

	Placebo N (%) or Median (IQR) *n* = 24	Treatment N (%) or Median (IQR) *n* =25	*p*
**IBS-SSS Items**			
Abdominal Pain V1	35 (27.5–65)	35 (25–55)	0.695
Abdominal Pain V2	50 (42.5–70)	20 (5–35)	0.001
Days with abdominal Pain in the last 10 V1	55 (35–80)	40 (20–70)	0.315
Days with abdominal Pain in the last 10 V2	55 (30–90)	30 (20–60)	0.032
Bloating V1	65 (40–82.5)	55 (50–75)	0.567
Bloating V2	62.5 (37.5–80)	40 (25–65)	0.021
Satisfaction bowel habits V1	22.5 (10–50)	25 (15–40)	0.951
Satisfaction bowel habits V2	30 (15–50)	45 (30–70)	0.035
Interference with daily activities V1	70 (47.5–90)	55 (40–90)	0.421
Interference with daily activities V2	55 (40–75)	40 (30–65)	0.118
**Clinical outcomes**			
IBS-SSS Score V1	240 (207.5–330)	240 (200–270)	0.250
IBS-SSS Score V2	265 (217.5–317.5)	195 (145–230)	0.007
IBSS-SSS Score variations (Delta V1–V2)	1.5 (−37.5–35)	50 (−5–75)	0.032
Responders (reduction 50 points IBS-SSS)	4 (16.7%)	13 (52%)	0.009
**IBS Subtypes**			
**IBS-C**	*n* = 2	*n* = 4	
IBS-SSS Score V2	155 (60–250)	180 (160–192.5)	1
Responders (reduction 50 points IBS-SSS)	1 (50%)	3 (75%)	0.540
**IBS-D**	*n* = 9	*n* = 8	
IBS-SSS Score V2	245 (220–305)	218.5 (185–260)	0.470
Responders (reduction 50 points IBS-SSS)	2 (22.2%)	3 (37.5%)	0.490
**IBS-M**	*n* = 13	*n* = 13	
IBS-SSS Score V2	310 (255–320)	190 (130–230)	0.005
Responders (reduction 50 points IBS-SSS)	1 (7.7%)	7 (53.9%)	0.011

**Abbreviations:** N: number; IQR: interquartile range; V1: visit 1; V2: visit 2; IBS-SSS: irritable bowel syndrome symptom severity score; IBS-C: irritable bowel syndrome with constipation; IBS-D: irritable bowel syndrome with diarrhea; IBS-M: irritable bowel syndrome with mixed bowel habits.

## Data Availability

Sequence reads of fecal microbiota analysis were deposited in the National Center for Biotechnology Information Sequence Read Archive (NCBI SRA; BioProject ID PRJNA852514).

## Supplementary Materials

The following are available online at https://www.mdpi.com/article/10.3390/nu14194208/s1, Figure S1: Beta diversity of the gut microbiota of IBS patients after geraniol (LAGS) or placebo treatment. PCoA based on Bray-Curtis dissimilarity between the genus-level profiles of IBS (A) or IBS-M (B) patients before (V1) and after 4 weeks (V2) of treatment with LAGS or placebo. Ellipses include 95% confidence area based on the standard error of the weighted average of sample coordinates. No significant differences were found (*p* > 0.1, Adonis). Figure S2: Compositional structure of the gut microbiota of IBS patients after geraniol (LAGS) or placebo treatment. Bar plots and pie charts showing the relative abundance of major phyla (A) and families (B) of IBS patients before (V1) and after 4 weeks (V2) of treatment with geraniol or placebo. Only taxa with relative abundance >0.1% in at least two samples were considered. Figure S3: Plasma cytokine and chemokine variations measured at V1 and V2 in the geraniol (LAGS) arm divided into IBS subtypes in comparison with placebo arms. Analytes were determined using a 13-plex human immunoassay Luminex^®^ kit. Graphs of cytokines quantifiable in plasma are shown. For all analytes, *p* > 0.1.
