# Supplementary figures and images for: Geraniol Treatment for Irritable Bowel Syndrome: A Double-Blind Randomized Clinical Trial
Source: Nutrients. 2022 Oct 10;14(19):4208. doi: 10.3390/nu14194208 (PMC9571173; doi:10.3390/nu14194208)

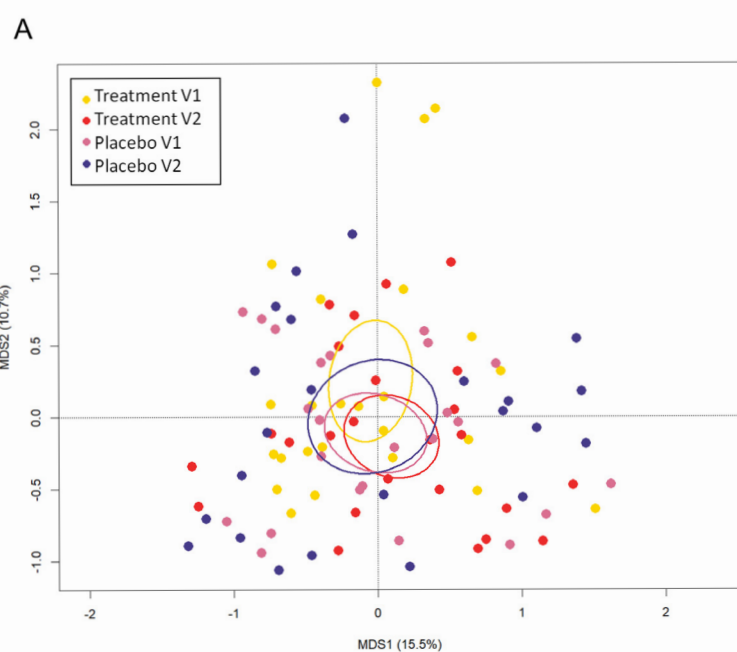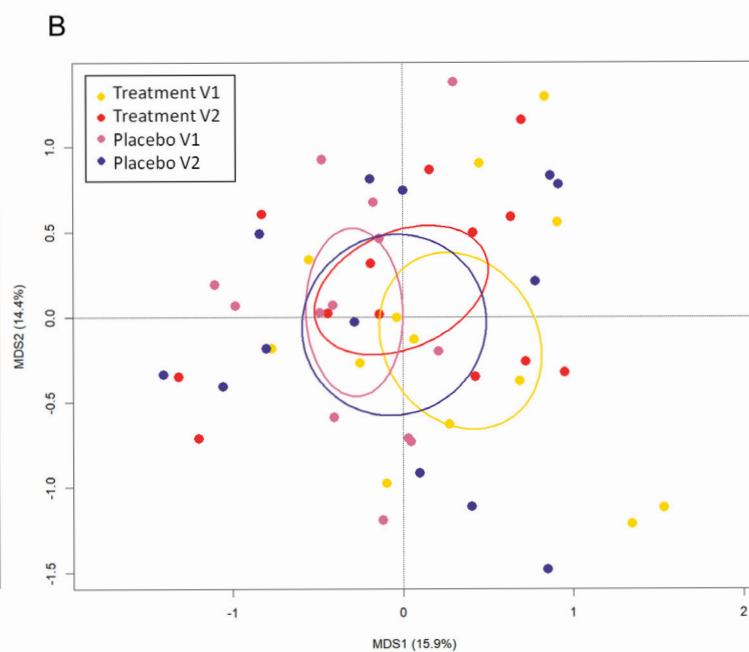

Figure S1

Supplement: Supplementary file 1 [file nutrients-14-04208-s001.zip › Figure S1.pdf]

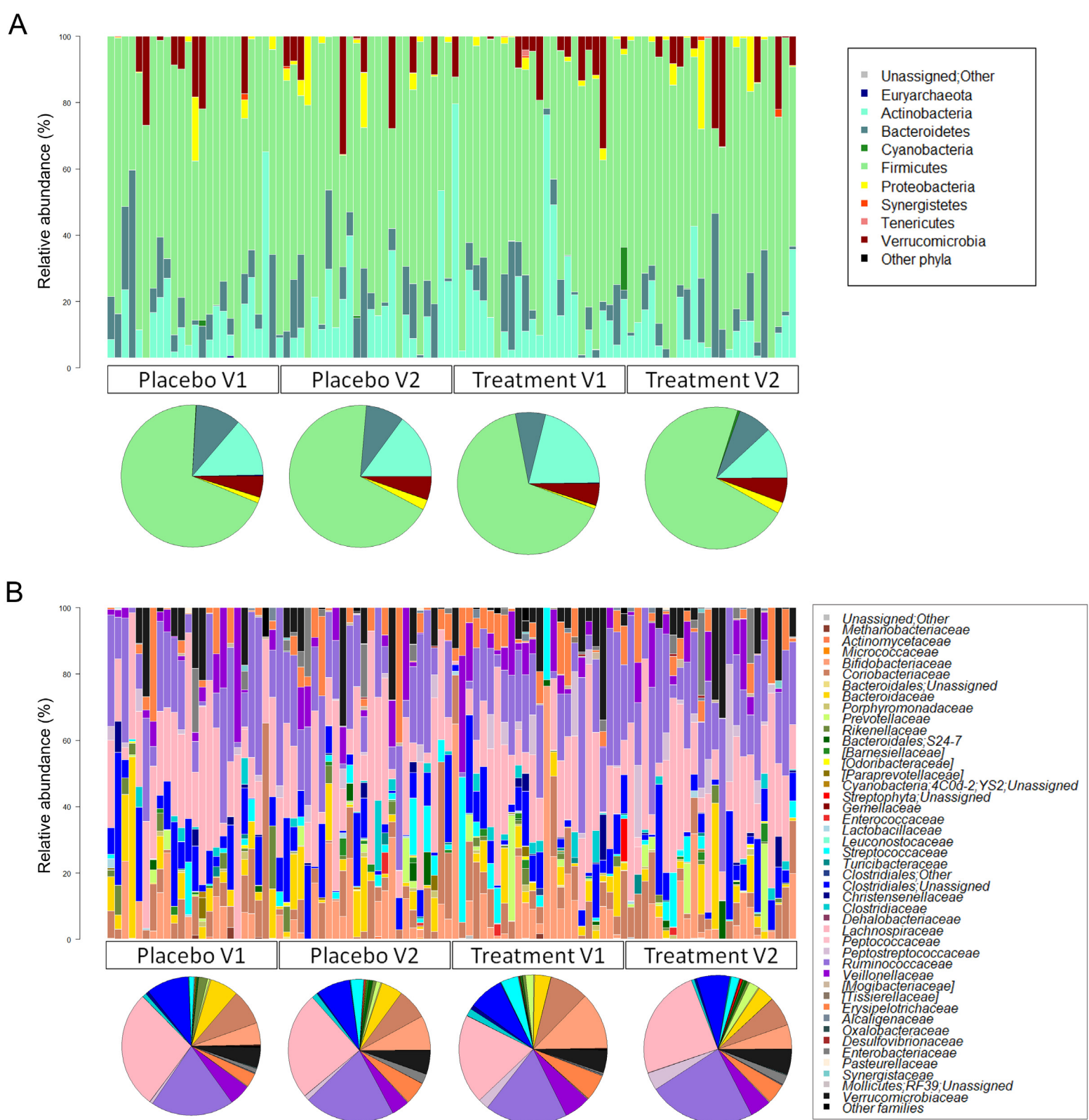

Figure S2

Supplement: Supplementary file 1 [file nutrients-14-04208-s001.zip › Figure S2.pdf]

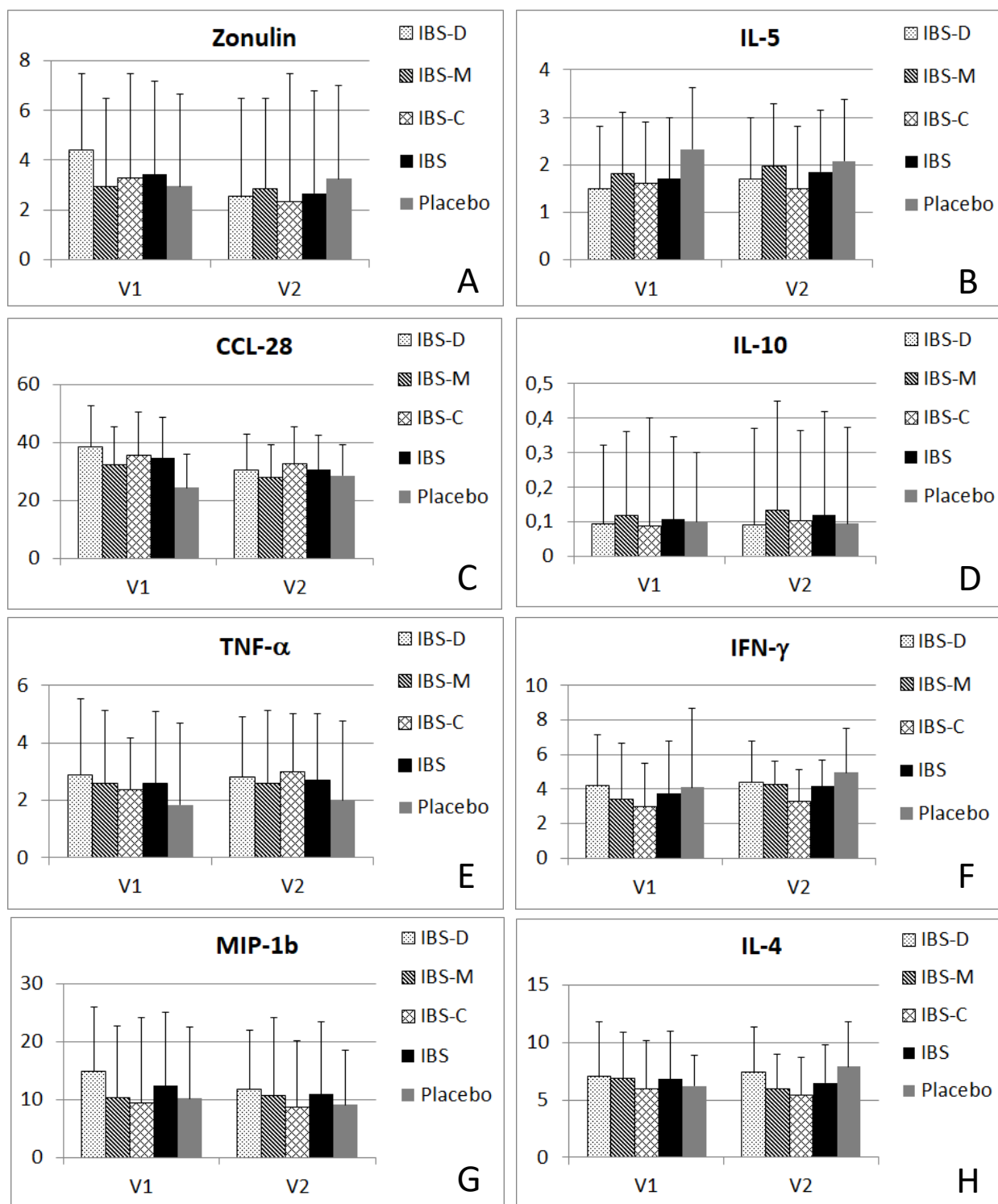

Figure S3

Supplement: Supplementary file 1 [file nutrients-14-04208-s001.zip › Figure S3.pdf]
